# The role of attention and health goals in nudging healthy food choice

**DOI:** 10.3389/fpsyg.2023.1270207

**Published:** 2023-10-19

**Authors:** Marleen Gillebaart, Stephanie S. A. H. Blom, Jeroen S. Benjamins, Femke de Boer, Denise T. D. De Ridder

**Affiliations:** ^1^Department of Social, Health and Organizational Psychology, Utrecht University, Utrecht, Netherlands; ^2^Department of Experimental Psychology, Utrecht University, Utrecht, Netherlands

**Keywords:** nudging, food choices, healthy eating goals, attention, experimental field study

## Abstract

**Introduction:**

Nudging is a promising intervention technique that supports people in pursuing their healthy eating goals. Recent research suggests that, despite previous assumptions, disclosure of the presence of a nudge does not compromise nudge effectiveness. However, it is unknown whether attention to a nudge affects nudge effects. We assessed the role of attention systematically, by examining explicit and implicit attention to nudges, while also exploring healthy eating goals as a potential moderator.

**Methods:**

Participants were assigned to a nudge (i.e., a shopping basket inlay with pictures of healthy items) or control condition (i.e., a shopping basket inlay with neutral pictures) and chose a snack in an experimental supermarket field study. Explicit and implicit attention (with a mobile eye-tracker) to nudges, healthiness of snack choice, and healthy eating goals were assessed.

**Results:**

Results showed that attention to the nudge did not hamper the nudge’s effect. Furthermore, individuals with strong healthy eating goals made healthier food choices in the nudge condition. Individuals with weak to non-existent healthy eating goals were not influenced by the nudge.

**Discussion:**

Findings are in line with the viewpoint that nudging does not by definition work ‘in the dark’, and suggests that nudges support people in adhering to their healthy eating goal.

## 1. Introduction

Employing ‘nudging’ as an intervention technique can make the desirable, healthier choice the easier choice (Thaler and Sunstein, 2008; Marteau et al., 2011; De Ridder et al., 2017). This approach for promoting people’s health behavior is in line with advice from the World Health Organization (WHO) stating that in order to combat the current obesity epidemic, healthier food choices should be made the easier choices (World Health Organization, 2021a,b). Nudges are defined as “any aspect of the choice architecture that alters people’s behavior in a predictable way without forbidding any options or significantly changing their economic incentives” (Thaler and Sunstein, 2008, p. 6). Recent meta-analyses confirm that nudging can stimulate healthy food choices (Arno and Thomas, 2016; Bucher et al., 2016; Broers et al., 2017; Cadario and Chandon, 2020). Importantly though, knowledge on the psychological premises and the boundary conditions of nudging remains limited (Marchiori et al., 2017; Szaszi et al., 2018; Harbers et al., 2020). Because of its potential and popularity, it is vital that we know how nudges operate in terms of the roles of people’s attention to nudges and their pre-existing (health) goals.

The nudge concept is derived from notions on dual process theories, roughly dividing human decision making into two types: ‘system 1’ and ‘system 2’ reasoning (e.g., Kahneman, 2003; Evans, 2008; Melnikoff and Bargh, 2018; though there continues to be debate on these theories, see for example Keren and Schul, 2009; Bago and De Neys, 2017; Melnikoff and Bargh, 2018). A core premise of nudge theory is that nudges do not work by combating individuals’ prevalent fast, automatic, and effortless system 1 reasoning (e.g., Gigerenzer, 2015), but rather by making use of this system by redirecting it towards more desirable choices. The common assumption that nudge effectiveness stems from its influence on behavior automatically, and therefore outside of the realm of attention, has however not been tested in an extensive and systematic manner (Marchiori et al., 2017). An unanswered question is whether nudging works exclusively works *via* individuals’ fast and automatic (also referred to as ‘system 1′) reasoning, as posited in the original introduction of the nudge concept (Thaler and Sunstein, 2008). If this would be the case, attending to a nudge would interfere with a nudge’s effectiveness, as it would imply a switch to more reflective (also referred to as ‘system 2′) reasoning. However, recent studies have suggested that nudge disclosure does not necessarily compromise effectiveness (Papies et al., 2014; Van Gestel et al., 2018; Huitink et al., 2020).

A few studies have assessed the effect of explicitly disclosing the presence of a nudge on a nudge’s effectiveness, showing that this does not decrease the nudge’s effect (e.g., Kroese et al., 2016; Cheung et al., 2019). For example, a field study in a hospital cafeteria found that placing fresh fruits at the front counter of a take-away food vendor increased sales of these products, irrespective of the presence of a disclosure message conveying the purpose of the nudge (Cheung et al., 2019). Similar findings have been reported in studies in which desirable choices other than healthy diet choices were nudged (e.g., Loewenstein et al., 2015; Steffel et al., 2016; Bruns et al., 2018). Explicit disclosure of a nudge thus does not appear to interfere with the nudging effect. However, disclosing the presence of a nudge need does not necessarily mean that individuals are paying attention to a nudge (Marchiori et al., 2017).

The few studies that do explicitly report on attention to nudges generally show that only a small number of people spontaneously notice a nudge when probed about their attention to the nudge (e.g., Kroese et al., 2016; Van Gestel et al., 2018; Blom et al., 2021). For instance, when asked if they noticed anything out of the ordinary in a supermarket, only 26% of participants reported to have perceived a nudge in a field study nudging healthy food choices (Blom et al., 2021). Importantly, attending to a nudge did not impede the nudging effect, as the nudge stimulated healthy food choice irrespective of whether participants reported to have noticed the nudge. In another field study nudging healthy food choices by using an inlay in a supermarket shopping cart (Huitink et al., 2020), attention to the nudge was probed by referring more specifically to the location of the nudge, namely by asking customers if they noticed anything different in their shopping cart. Here, about 73% of customers noticed the nudge, a much higher proportion than reported in other studies (e.g., Van Gestel et al., 2018; Blom et al., 2021), which may be due to the specificity of the question probing nudge attention. Importantly, attention to the nudge did not hamper effectiveness but rather proved a boundary condition as only nudge-attending customers purchased the nudged products. Other studies (Papies et al., 2014; Van der Laan et al., 2017) likewise suggest that attention to a nudge might be more prevalent than assumed based on studies probing attention to nudges in a more general manner. In an online study for instance, participants paid more implicit attention, as measured by eye-tracking, to a banner displaying a health nudge vs. a control banner (Van der Laan et al., 2017). While this study shows that eye-tracking can be suitable to measure implicit attention to a nudge, the role of implicit attention in the nudge’s effectiveness was not examined.

Taken together, recent research suggests that attention to a nudge as well as explicit disclosure of the presence of a nudge does not hamper nudge effectiveness. This suggests that contrary to what would be expected based on the theoretical premises of nudging, nudging does not solely hinge on system 1 decision making. In fact, recent research shows that nudges can also be effective under system 2 decision making where people were encouraged to reflect on their choice (Van Gestel et al., 2020). However, the role of attention in nudging remains to be examined systematically, as indicated by the discrepancy in findings of studies employing different measures of attention to nudges. This underlines the importance of using specific and detailed questions to probe (explicit) attention to a nudge, as well as the importance of corroborating self-report measures by use of implicit measures of attention like eye-tracking.

In addition to attention as an important factor in how nudges operate, research into nudging should also consider whether nudges support people’s in pursuing their pre-existing (health) goals, or whether they affect behavior regardless of any pre-existing goals. Previous studies have indicated that pre-existing preferences can be considered a relevant boundary condition for nudging (Sunstein, 2017; De Ridder et al., 2022). The current study therefore also takes into account whether the nudged behavior aligns with an individuals’ goals or preferences. The fact that most people do not meet the recommendations for a healthy diet (De Ridder et al., 2017), despite their healthy eating goals (De Ridder et al., 2014), makes examination of the role of healthy eating goals in nudging healthy food choice highly relevant. Recent research suggests that nudging might be ineffective when individuals already have a strong preference for the nudged behavior (Venema et al., 2019, 2020). However, it should be noted that these studies examined nudging in lab studies, in choice settings which were less complex and tempting than in real life. In more complex and tempting real life settings, nudges may prove effective in supporting individuals only when they align with people’s healthy eating goals (De Ridder et al., 2022).

### 1.1. Current study

The current study examines the role of attention and health goals in nudging the purchase of healthy food options in an experimental field study in a supermarket, with healthiness of snack choice as the main outcome measure. Stimulating healthy snack choice is important as snacking tends to involve the consumption of food of poor nutritional quality (Larson and Story, 2013), and is associated with unhealthy diet and obesity (Forslund et al., 2005). Because snacks are predominantly consumed at home (e.g., Kerr et al., 2010; Myhre et al., 2015; Schlinkert et al., 2020), decisions made in the supermarket largely define the snacks people end up consuming (Papies et al., 2014), deeming the supermarket a relevant location to nudge healthier snack choices.

We hypothesized that nudge effectiveness would not be hindered by attention to the nudge, hence that the nudge is also effective when people pay attention to the nudge – either implicitly or explicitly. As it has been reasoned that nudging might only be effective when the nudged behavior aligns with people’s goals (De Ridder et al., 2022), we also examined the role of healthy eating goals as a potential moderator of the nudging effect. Specifically, we explored whether the nudge only stimulates healthier food choices for people with a healthy eating goal. If the goal to eat healthily is weak or non-existent, we did not expect that the presence of a nudge would result in a healthy choice regardless of whether people pay attention to the nudge. This study furthers the rather limited knowledge on the psychological factors involved in nudging (Marchiori et al., 2017), by examining the role of attention and healthy eating goals in nudging in a complex, real-life choice environment.

## 2. Materials and methods

### 2.1. Study design and sample size

**Figure 1:**
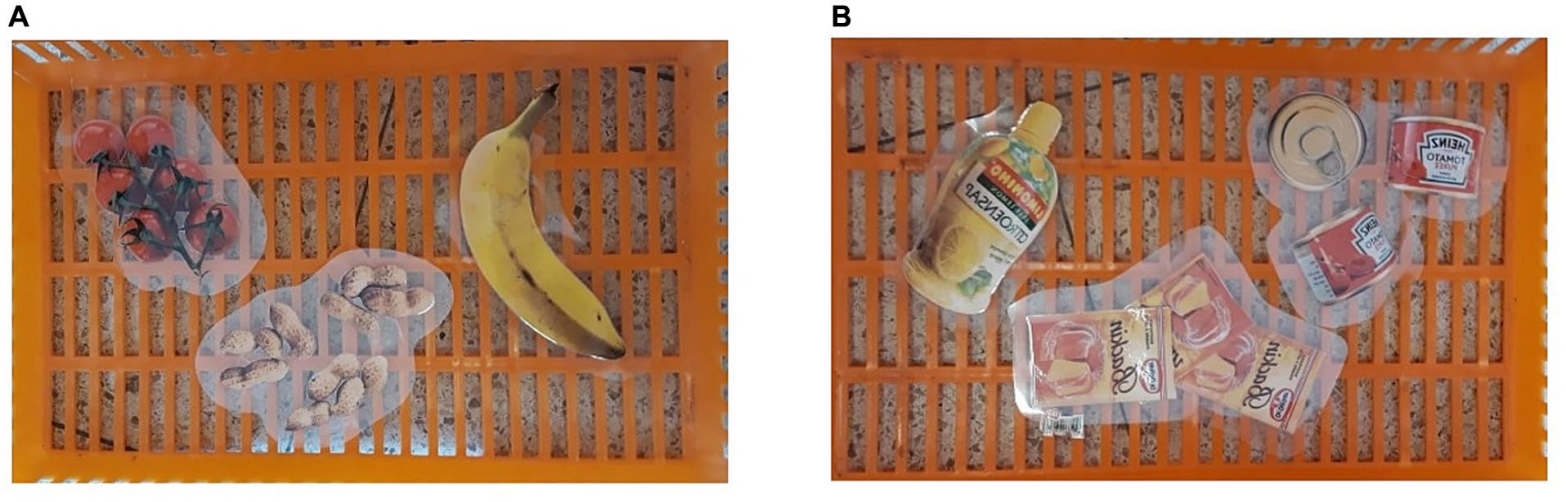
Pictures used in the nudge condition **(A)** and the control condition **(B)**.

This study examined healthy food choice by means of an experimental field study in a supermarket. We compared the effect of a nudge condition, in which images of healthy food products were depicted in the shopping basket participants use during the task, to a control condition in which the images depicted neutral food products. Participants were randomly assigned to either the nudge or control condition. Plasticized pictures were placed in the shopping baskets participants used for their shopping. In the experimental nudge condition, healthy food products were depicted: a yellow banana, brown peanuts, and red tomatoes (Figure 1A). In the control condition, neutral food products were depicted: a yellow bottle of lemon juice, three brown bags of baking powder, and three red cans of tomato paste (Figure 1B). Colors of the pictures in both conditions were similar.

Healthiness of snack choice was the main dependent variable. Explicit attention to the pictures and healthy eating goals were measured by use of questionnaires. A mobile eye tracker was used to measure participants’ eye movements during the task in order to determine their fixations on the pictures depicted in the shopping basket, i.e., implicit attention to the pictures.

As no previous studies examined the effect of implicit attention in combination with nudging on food purchases in a supermarket field study, the required sample size was initially based on sample sizes previously used in field studies employing mobile eye-tracking (Otterbring et al., 2014; Wästlund et al., 2015). In line with these studies, we aimed to recruit approximately 50 participants per condition, hence 100 participants in total. Running a sensitivity analysis in G*Power 3.1 (α = 0.05, power = 80%, *N* = 97, number of predictors = 5) for a multiple regression analysis indicated that we would be able to detect a difference of small to medium effect size between the two experimental conditions in our experimental design, effect size f^2^ = 0.08. The study was approved by the Ethics Review Board of the Faculty of Social and Behavioral Sciences at Utrecht University, filed under number FETC19-032.

### 2.2. Participants

Participants were Dutch speaking customers doing their grocery shopping at a supermarket in a large city in the Netherlands. Upon entering the supermarket, all customers were approached and asked whether they were willing to participate in a study. Participants were then included if they were at least 18 years old, did their grocery shopping unaccompanied, did not wear eyeglasses, did not have severely drooping eyelids, and were not wearing mascara. The latter three criteria were related to the ability to correctly wear and use the mobile eye tracker. In total, 107 participants took part in this study. Twelve participants had to be excluded because they did not correctly follow the instructions. Of these participants, eleven were excluded because they chose more than one snack, and one because of asking someone else in the supermarket for advice about which snack to choose.

This resulted in a final sample of ninety-five participants (49 female), with ages ranging from 18 to 66 (*M*_age_ = 35.96 years old, *SD*_age_ = 12.79 years). The majority (77%) had attained high levels of education, 20% attained middle levels of education, and 3% attained low levels of education, according to the European Qualifications Framework (European Commission, n.d.). Based on their self-reported weight and height, the majority of participants (71%) had normal weight, while 29% had overweight. Three participants did not answer some of the questions, but were included in the analysis of the other variables. For the eye tracking data only, fourteen extra participants were excluded, due to a failure of the eye tracker glasses to record their eye fixations (8 participants) or because the participant also did their own groceries during their snack shopping task (6 participants).

### 2.3. Procedure

Data collection was done on weekdays over the course of 2 weeks, between 10 AM and 4 PM. When customers entered the supermarket, they were invited to participate in an experiment for which they would receive a free product and a present. If the customer was interested, it was explained that: “*We would like to investigate how people make decisions in the supermarket*.” Participants were told that they would have to wear a mobile eye tracker and carry a shopping basket. After signing the informed consent, the eye tracker was installed and calibrated. Then, the task was explained as follows: “*Buy a snack of maximum two euros and fifty cents. A snack is something you can eat now or later in between your three main meals*” (Hess et al., 2016). Following this introduction, participants were provided with the shopping basket with a plasticized picture in it, entered the supermarket, picked the snack of their choice and walked to the checkout line. There, the experimenter was waiting for them, removed the eye tracker and paid for the chosen product. Before leaving the supermarket, participants were asked to fill out a questionnaire, after which they were debriefed. Participants were asked not to tell other people about the goal of the experiment. Upon finalizing the experiment, participants received their chosen snack and an additional present (i.e., pens and notepads). They could leave their email address if they wanted to participate in a raffle for a gift coupon of 20 euros.

### 2.4. Measures

#### 2.4.1. Healthiness snack choice

To examine whether the nudge was effective in stimulating healthier snack choices, we calculated the Nutri-Score (Chantal et al., 2017) of the chosen snacks. These scores were subsequently recoded into a numerical score for the analyses such that a higher score reflected a healthier snack choice (i.e., Nutri-score E = 1, D = 2, C = 3, B = 4, A = 5). For unprocessed fruits or vegetables, no Nutri-score can be calculated. These products were therefore manually scored, and received a score of 5.

It was verified whether snack choices that are objectively (i.e., based on Nutri-score) healthier were also considered healthier choices by participants. The perceived healthiness of participants’ snack choice was measured using the statement *“I think this product is a healthy choice,”* to be answered with yes or no. A Pearson’s correlation showed that healthiness snack choice as indicated by the Nutri-score was positively associated with perceived healthiness snack choice, *r* = 0.57, *p* < 0.001. This indicates that participants’ perception of healthiness of their chosen snack was accurate.

#### 2.4.2. Explicit attention

Explicit attention to the pictures was measured with three items. First, we asked “*Have you looked at the pictures in the shopping basket?*,” to be answered with yes or no. Second, we asked “*How extensively have you looked at the pictures in the shopping basket?*” to be answered on a scale from 1 (*not extensively at all*) to 7 (*very extensively*) (based on Papies et al., 2014). Third, an open question was added in which we asked participants to write down the depicted products in the shopping basket. They could write down the products they remembered, or answer that they did not know. The answers to this question were subsequently coded into 0 (*I do not know/false*), 1 (*remembered one product correctly*), 2 (*remembered two products correctly*), and 3 (*remembered all three products correctly*).

#### 2.4.3. Implicit attention

A Tobii Pro Glasses 2 mobile eye tracker was used to measure gaze behavior of participants at 50 Hz. The glasses’ head unit of the eye tracker was fit on the participants’ head like normal glasses. One point calibration of the system was performed (in line with the manufacturers procedure) for each participant individually by use of a calibration card. Raw eye tracking data was preprocessed and coded with respect to fixations by use of GazeCode (Benjamins et al., 2018) in Matlab. Fixations were operationalized by use of a slow-phase classifier (Hooge and Camps, 2013; Benjamins et al., 2018). Fixations on the pictures in the shopping basket as well as the total number of fixations during the shopping task were used to calculate the percentage of fixations on the pictures.

#### 2.4.4. Healthy eating goal

To measure whether participants considered healthy eating important, they rated the statement “*I think it is important to eat healthily*,” on a scale from 1 (*totally disagree*) to 7 (*totally agree*) (Van Gestel et al., 2018).

#### 2.4.5. Control measures

The following control measures were included: hunger, BMI, general snacking habit, and store familiarity. These measures are described in more detail in the Supplementary material.

### 2.5. Statistical analyses

We first checked whether randomization across experimental conditions was successful by comparing the control measures of participants in the two different conditions. Subsequently, we examined whether explicit and implicit attention differed based on condition by means of a Mann–Whitney U-tests. We then conducted multiple regression analyses including experimental condition and interactions between condition and both types of attention, as well as healthy eating goal, to test whether nudges were effective when customers paid attention to them, and to examine the role of healthy eating goals in nudging.

## 3. Results

### 3.1. Descriptive analyses and randomization checks

Means and standard deviations of variables of interest for the whole sample and per condition are presented in Table 1.

**Table 1:** Means and standard deviations for variables of interest, in total and per condition.

	Full sample (*N* = 95)	Nudge condition (*N* = 44)	Control condition (*N* = 51)
	*M* (SD)	*M* (SD)	*M* (SD)
Explicit attention			
Extent of looking	2.28 (1.60)	2.77 (1.83)	1.86 (1.25)
Item memory	0.45 (0.54)	0.66 (0.57)	0.27 (0.45)
Implicit attention	2.74 (6.59)	1.53 (3.47)	1.03 (1.16)
Health goal importance	5.76 (1.02)	5.91 (0.74)	5.63 (1.20)
Healthiness snack choice	2.59 (1.43)	2.66 (1.31)	2.53 (1.54)

#### 3.1.1. Randomization checks

Independent samples *t*-tests indicated that age (*p* = 0.711), BMI (*p* = 0.367), general snack habit (*p* = 0.896), store familiarity (*p* = 0.278), and hunger (*p* = 0.074) were not significantly different across conditions. Gender (Fisher exact test *p* = 0.540) and educational level (Pearson Chi-square test *p* = 0.723) were not significantly different across conditions either, indicating successful randomization.

#### 3.1.2. Attention

We first examined whether implicit attention (percentage of fixations on the pictures) was associated with explicit self-reported attention to the pictures. Participants who reported to have looked at the pictures had higher implicit attention scores (*M* = 1.69, SD = 3.20), compared to those who reported not to have looked at the pictures (*M* = 0.66, SD = 1.09, *U* = 528.00, *p* = 0.009). Furthermore, the more extensively participants reported to have looked at the pictures, the higher their implicit attention score (*r* = 0.22, *p* = 0.050,). Lastly, the more pictures participants recalled correctly, the higher their implicit attention score (*r* = 0.27, *p* = 0.017). Next, we examined whether the three explicit attention measures were positively associated with each other, which was the case. Participants who reported to have looked at the pictures also reported to have looked more extensively at the pictures (*r* = −0.63, *p* < 0.001), and remembered more pictures correctly (*r* = −0.47, *p* < 0.001). Furthermore, the more extensive participants reported to have looked at the pictures, the more pictures they remembered correctly (*r* = 0.54, *p* < 0.001). Together, these findings suggest substantive associations within and between explicit and implicit measures of attention, indicating that self-reported attention is backed up by unbiased implicit attention to the nudge.

### 3.2. Nudge condition and attention

We assessed whether participants paid more explicit attention in the nudge compared to the control condition. A Fisher’s exact test revealed that 75.0% of participants in the nudge condition reported to have looked at the displayed pictures, while in the control condition only 43.1% of participants reported to have looked at the displayed pictures, *p* = 0.002, *Phi* = 0.32. A Mann–Whitney U-test showed that participants in the nudge condition also looked at the pictures more extensively (*M* = 2.77, SD = 1.83), than participants in the control condition (*M* = 1.86, SD = 1.25), *U* = 1456.50, *p* = 0.008. Moreover, a Mann–Whitney U-test confirmed that the number of correctly remembered displayed pictures was significantly higher for participants in the nudge condition (*M* = 0.66, SD = 0.57), compared to those in the control condition (*M* = 0.27, SD = 0.45), *U* = 1516.50, *p* < 0.001. Finally, a Mann–Whitney U-test demonstrated that participants in the nudge condition (*M* = 1.57, SD = 3.51) did not pay more *implicit* attention to the nudge compared to the control condition (*M* = 0.98, SD = 1.17), *U* = 849.00, *p* = 0.777.

Taken together, the nudge (vs. control) condition was associated with more explicit, but not implicit attention to the pictures displayed in the shopping cart. We consider the reported extensivity of looking at the pictures the most informative measure of explicit attention. We will therefore employ this measure in the main analyses.

### 3.3. Main analyses

#### 3.3.1. Influence of nudge condition, explicit attention, and healthy eating goal on snack choice

To examine the role of explicit attention in nudge effectiveness, we conducted a multiple hierarchical regression analysis with centralized healthy eating goal as a moderator. In the first step, experimental condition (0 = control, 1 = nudge), explicit attention, and healthy eating goal were included. In the second step, the interaction terms condition*explicit attention and condition*healthy eating goal were added as predictors of the dependent variable healthiness of snack choice (Nutri-score). Both the model including only main effects and the model including interaction effects were not significant, *F* (3, 91) = 0.07, *p* = 0.98, *R*^2^ = 0.00, *Adjusted R*^2^ = −0.03 and *F* (5, 89) = 1.47, *p* = 0.208, *R*^2^ = 0.08, *Adjusted R*^2^ = 0.02, respectively.

Neither condition, explicit attention, healthy eating goal, or the interaction term between experimental condition and attention did significantly predict healthy snack choice. The absence of an interaction effect between condition and attention indicates that the nudge did not stimulate healthier snack choice, irrespective of whether the participant paid attention to the displayed pictures or not. The only significant predictor of healthy snack choice was the interaction term between condition and healthy eating goal, *β = 0.*31, *t* = 2.65, *p* = 0.010. Simple slope tests were performed to probe this interaction for one SD above and below the mean of healthy eating goal (Aiken et al., 1991). The simple slope for a high healthy eating goal was significant, *t* (94) = 2.43, *β* = 0.96*, p* = 0.017, while the simple slope for a low healthy eating goal was not, *t* (94) = −1.64, *β* = −0.85, *p* = 0.104. This implicates that for people with a strong healthy eating goal the nudge resulted in healthier snack choices compared to people with weaker healthy eating goals (see the Supplementary materials for a visual depiction of the simple slope analysis). Hence, healthy eating goals indeed moderated the nudging effect in line with expectations.

In order to examine whether the interaction between nudging and a healthy eating goal was further qualified by an interaction with attention (e.g., people with a stronger healthy eating goal paying more attention to the nudge), a second multiple regression analysis was performed including this three-way interaction term in the second step. However, this three-way interaction term did not predict healthiness snack choice (*p* = 0.935), while the interaction term between experimental condition and healthy eating goal remained the only significant predictor of healthiness snack choice (*p* = 0.013). This indicates that attention, as expected, did not hamper the nudging effect (see Table 2).

**Table 2:** Regression model for explicit attention including interactions with healthiness snack choice as dependent variable.

	*b (SE)*	*β*	95% CI *b*	*t*	*p*
Constant	2.48 (0.21)		[2.07, 2.90]	11.80	< 0.001
Condition	0.05 (0.31)	0.02	[−0.56, 0.67]	0.17	0.867
Attention	−0.04 (0.16)	−0.04	[−0.36, 0.28]	−0.23	0.819
Healthy eating goal	−0.22 (0.17)	−0.16	[−0.56, 0.11]	−1.34	0.185
Condition X attention	0.09 (0.20)	0.07	[−0.31, 0.48]	0.43	0.672
Condition X healthy eating goal	0.89 (0.34)	0.31	[0.22, 1.56]	2.65	0.010

*N* = 95. Condition is coded as 0 = control, 1 = nudge.

#### 3.3.2. Influence of nudge condition, implicit attention, and healthy eating goal on snack choice

To examine the role of implicit attention in nudge effectiveness, we conducted another hierarchical regression analysis with centralized healthy eating goal as a moderator. In the first step, experimental condition (0 = control, 1 = nudge), implicit attention, and healthy eating goal were included. In the second step, the interaction terms condition*implicit attention and condition*healthy eating goal were added as predictors of the dependent variable healthiness of snack choice (Nutri-score). Both the model including only main effects and the model including interaction effects were not significant, *F* (3, 77) = 0.14, *p* = 0.94, *R*^2^ = 0.00, *Adjusted R*^2^ = −0.03 and *F* (5, 75) = 1.77, *p* = 0.129, *R*^2^ = 0.11, *Adjusted R*^2^ = 0.05, respectively.

The absence of an interaction effect between condition and attention again indicates that the nudge did not stimulate healthier snack choice, irrespective of whether the participant paid attention to the displayed pictures or not. As can be seen in Table 3, the interaction term between condition and health goal was the only significant predictor, *β = 0.*29, *t* = 2.21, *p* = 0.030. Simple slope tests were performed to probe this interaction for one SD above and below the mean of healthy eating goal (Aiken et al., 1991). The simple slope for high health goal was not significant but did show a marginally significant trend, *t* (80) = 1.93, *β* = 0.85, *p* = 0.058. The simple slope for low health goal was not significant and did not show a trend, *t* (80) = −1.51, *β* = −0.76, *p* = 0.134. This suggests that, even though both simple slopes did not reach significance, for people with a stronger health goal the nudge (vs. control) induced healthier snack choices.

**Table 3:** Regression model for implicit attention including interactions with healthiness snack choice as dependent variable.

	*b (SE)*	*β*	95% CI *b*	*t*	*p*
Constant	2.46 (0.22)		[2.03, 2.90]	11.26	< 0.001
Condition	0.06 (0.31)	0.02	[−0.56, 0.68]	0.20	0.844
Implicit attention	−0.32 (0.19)	−0.60	[−0.69, 0.53]	−1.71	0.092
Healthy eating goal	−0.16 (0.17)	−0.12	[−0.50, 0.18]	−0.94	0.349
Condition X implicit attention	0.29 (0.20)	0.52	[−0.10, 0.69]	1.49	0.142
Condition X healthy eating goal	0.76 (0.34)	0.29	[0.08, 1.44]	2.22	0.30

*N* = 81. Condition is coded as 0 = control, 1 = nudge.

## 4. Discussion

The present study examined the role of attention and healthy eating goals in nudging healthy food choice. To date, discrepant findings have been reported in the literature with respect to the role of attention to nudges in nudges’ effectiveness, which can possibly be explained by the variety of methods used to probe attention, including relying on merely disclosing the presence of a nudge. Therefore, we measured explicit attention by use of specific and detailed questions, and implicit attention by use of eye-tracking in order to substantiate the explicit attention measures.

With respect to attention to the nudge, participants in the nudge condition paid more explicit, but not implicit attention to the pictures displayed in the shopping basket (i.e., the nudge) than participants in the control condition did [in line with Van der Laan et al. (2017), Papies et al. (2014), and Huitink et al. (2020)], a difference which showed on all three measures of explicit attention. In fact, 75% of participants in the nudge condition reported to have paid attention to the nudge. This is a much larger proportion than reported in studies probing spontaneous nudge awareness (e.g., Van Gestel et al., 2018; Blom et al., 2021), but it concurs with recent results of a study probing attention to a nudge with more specific questions (Huitink et al., 2020). Importantly, although the implicit attention measure was correlated with the explicit attention measures, there were no differences in implicit attention between the nudge and control condition, emphasizing that the specificity of the questions asked is an important factor when assessing aspects of attention. Together, the findings indicate that attention to nudges is more common than previously assumed, but that this attention is especially revealed when people are being asked about specific aspects of the nudge.

It was hypothesized that attention to the nudge would not impede nudge effectivity. There was no main effect of nudging on healthiness food choice, and no moderation of nudging by attention. Hence, the nudge was not effective in stimulating healthier food choices in the supermarket setting, irrespective of attention paid to the nudge. This finding suggests that nudge effectiveness does not rely on whether people are paying attention to a nudge. However, the nudge did stimulate healthy food choice for individuals with strong healthy eating goals, but not for participants with weak or nonexistent healthy eating goals. In fact, the latter group seems to show the opposite effect, although not statistically significant. The finding that healthy eating goals played a relevant role in nudge effectiveness concurs with the notion that pre-existing preferences are a relevant boundary condition for nudging (Sunstein, 2017; De Ridder et al., 2022). This moderation was not further qualified by an interaction with attention: individuals with strong healthy eating goals chose healthier snacks when nudged, irrespective of explicit attention. This indicates again that, as hypothesized, attention did not hinder the nudge’s effect and replicates previous research showing that nudges remain effective when individuals spontaneously notice a nudge (e.g., Van Gestel et al., 2018; Blom et al., 2021). It also concurs with the finding that enhancing system 2 reasoning does not render nudging ineffective (Van Gestel et al., 2020).

The finding that the nudge was only effective for individuals with goals that aligned with the nudge corresponds with the notion that a nudge would serve “the declared self-interests of those being nudged” (Hansen, 2016, p. 158) by supporting them in adhering to their goals. However, our findings appear to contrast with previous research indicating that nudges might be ineffective when individuals already have a strong preference for the nudged behavior (Venema et al., 2019, 2020). A possible explanation for this apparent discrepancy may be that the current study examined preferences and nudging in a real-life food choice setting with an abundance of potential temptations that do not align with healthy eating goals. This context probably makes it more difficult for individuals with healthy eating goals to act upon their goals, thus leaving more room for impact of a nudge supporting their goals as compared with less realistic and less tempting settings.

A limitation of the current study is that participants made only one food choice, and especially considering the supermarket setting, future research should also examine whether a nudge would affect multiple food choices. Another limitation may lie in the fact that participants made their choice while wearing a mobile eye-tracker, creating a more artificial supermarket experience than usual. Nevertheless, the mobile eye-tracker still allowed participants to move freely in a real supermarket environment during the study.

The current study’s results are particularly relevant with respect to the ongoing debate on the legitimacy of nudging. Nudges have been accused of operating ‘in the dark’ (Bovens, 2009) and, based on this notion, could be regarded as manipulative and undermining people’s freedom of choice (e.g., Bovens, 2009; Blumenthal-Barby and Burroughs, 2012; Grüne-Yanoff, 2012; Hansen and Jespersen, 2013; Gold et al., 2020). To date, this debate has largely relied on hypothetical situations and ethical arguments (De Ridder et al., 2022), though a recent line of empirical studies suggests that autonomy is not threatened in nudging contexts (Michaelsen et al., 2021; Wachner et al., 2021). The results of the current study further indicate that nudging does not necessarily compromise autonomy but rather may support it: the nudge was only effective for participants with healthy eating goals, indicating that the nudge contributed to choice-autonomy by supporting people to choose in line with their personal preferences while they were aware of the presence of the nudge (Schmidt and Engelen, 2020; De Ridder et al., 2022). Moreover, the absence of a hindering effect of attention indicates that nudges may operate in the dark, but also in the bright light of day.

## 5. Conclusion

The current study systematically examined the role of attention and healthy eating goals in nudging in a complex, real-life choice environment. Our findings show that nudging was only effective for individuals whose goals aligned with the nudged behavior. Attention did not hinder this nudging effect. These results advance the rather limited knowledge on psychological factors involved in nudging (Marchiori et al., 2017) by shedding light on the role of attention and personal goals in nudge effectiveness, thereby contributing to the ongoing debate on the legitimacy of nudging as an intervention technique.

## Data availability statement

The raw data supporting the conclusions of this article will be made available by the authors, without undue reservation.

## Ethics statement

The studies involving humans were approved by the Ethics Review Board of the Faculty of Social and Behavioral Sciences at Utrecht University. The studies were conducted in accordance with the local legislation and institutional requirements. The participants provided their written informed consent to participate in this study.

## Author contributions

MG: Conceptualization, Data curation, Formal analysis, Methodology, Project administration, Supervision, Writing – original draft, Writing – review & editing. SB: Data curation, Formal analysis, Writing – original draft. JB: Conceptualization, Formal analysis, Writing – original draft. FB: Data curation, Formal analysis, Methodology, Writing – original draft. DR: Conceptualization, Data curation, Formal analysis, Funding acquisition, Methodology, Project administration, Resources, Supervision, Writing – original draft, Writing – review & editing.

## References

[ref1] Aiken L. S. West S. G. Reno R. R. (1991). Multiple regression: testing and interpreting interactions. Thousand Oaks, California: Sage.

[ref2] Arno A. Thomas S. (2016). The efficacy of nudge theory strategies in influencing adult dietary behaviour: a systematic review and meta-analysis. BMC Public Health 16:676. doi: 10.1186/s12889-016-3272-x 27475752 PMC4967524

[ref3] Bago B. De Neys W. (2017). Fast logic?: examining the time course assumption of dual process theory. Cognition 158, 90–109. doi: 10.1016/j.cognition.2016.10.014 27816844

[ref4] Benjamins J. S. Hessels R. S. Hooge I. T. (2018). “GazeCode: open-source software for manual mapping of mobile eye-tracking data” in proceedings-ETRA 2018 (Association for Computing Machinery (ACM).)

[ref5] Blom S. S. Gillebaart M. De Boer F. van der Laan N. De Ridder D. T. (2021). Under pressure: nudging increases healthy food choice in a virtual reality supermarket, irrespective of system 1 reasoning. Appetite 160:105116. doi: 10.1016/j.appet.2021.105116 33450297

[ref6] Blumenthal-Barby J. S. Burroughs H. (2012). Seeking better health care outcomes: the ethics of using the “nudge”. Am. J. Bioeth. 12, 1–10. doi: 10.1080/15265161.2011.634481 22304506

[ref7] Bovens L. (2009). “The ethics of nudge” in Preference change: Approaches from philosophy, economics and psychology. eds. Grüne-Yanoff T. Hansson S. O. (New York: Springer), 207–219.

[ref8] Broers V. J. De Breucker C. Van den Broucke S. Luminet O. (2017). A systematic review and meta-analysis of the effectiveness of nudging to increase fruit and vegetable choice. Eur. J. Public Health 27, 912–920. doi: 10.1007/978-90-481-2593-7_10 28655176

[ref9] Bruns H. Kantorowicz-Reznichenko E. Klement K. Jonsson M. L. Rahali B. (2018). Can nudges be transparent and yet effective? J. Econ. Psychol. 65, 41–59. doi: 10.1016/j.joep.2018.02.002

[ref10] Bucher T. Collins C. Rollo M. E. McCaffrey T. A. De Vlieger N. Van der Bend D.. (2016). Nudging consumers towards healthier choices: a systematic review of positional influences on food choice. Br. J. Nutr. 115, 2252–2263. doi: 10.1017/S0007114516001653 27185414

[ref11] Cadario R. Chandon P. (2020). Which healthy eating nudges work best? A meta-analysis of field experiments. Mark. Sci. 39, 465–486. doi: 10.1287/mksc.2018.1128

[ref12] Chantal J. Hercberg S. World Health Organization (2017). Development of a new front-of-pack nutrition label in France: the five-colour Nutri-score. Public Health Panorama 3, 712–725.

[ref13] Cheung T. T. Gillebaart M. Kroese F. M. Marchiori D. Fennis B. M. de Ridder D. T. D. (2019). Cueing healthier alternatives for take-away: a field experiment on the effects of (disclosing) three nudges on food choices. BMC Public Health 19:974. doi: 10.1186/s12889-019-7323-y 31331307 PMC6647265

[ref14] De Ridder D. Adriaanse M. Evers C. Verhoeven A. (2014). Who diets? Most people and especially when they worry about food. Appetite 80, 103–108. doi: 10.1016/j.appet.2014.05.011 24845781

[ref15] De Ridder D. Kroese F. Evers C. Adriaanse M. Gillebaart M. (2017). Healthy diet: health impact, prevalence, correlates, and interventions. Psychol. Health 32, 907–941. doi: 10.1080/08870446.2017.1316849 28447854

[ref16] De Ridder D. Kroese F. van Gestel L. (2022). Nudgeability: mapping conditions of susceptibility to nudge influence. Perspect. Psychol. Sci. 17, 346–359. doi: 10.1177/1745691621995183 34424801 PMC8902020

[ref17] Evans J. S. B. T. (2008). Dual-processing accounts of reasoning, judgment, and social cognition. Annu. Rev. Psychol. 59, 255–278. doi: 10.1146/annurev.psych.59.103006.093629 18154502

[ref18] Forslund H. B. Torgerson J. S. Sjöström L. Lindroos A. K. (2005). Snacking frequency in relation to energy intake and food choices in obese men and women compared to a reference population. Int. J. Obes. 29, 711–719. doi: 10.1038/sj.ijo.0802950 15809664

[ref19] Gigerenzer G. (2015). On the supposed evidence for libertarian paternalism. Rev. Philos. Psychol. 6, 361–381. doi: 10.1007/s13164-015-0248-1 26213590 PMC4512281

[ref20] Gold N. Lin Y. Ashcroft R. Osman M. (2020). ‘Better off, as judged by themselves’: do people support nudges as a method to change their own behavior? Behav. Public Policy 7, 25–54. doi: 10.1017/bpp.2020.6

[ref21] Grüne-Yanoff T. (2012). Old wine in new casks: libertarian paternalism still violates liberal principles. Soc. Choice Welf. 38, 635–645. doi: 10.1007/s00355-011-0636-0

[ref22] Hansen P. G. (2016). The definition of nudge and libertarian paternalism: does the hand fit the glove? Eur. J. Risk Regul. 7, 155–174. doi: 10.1017/S1867299X00005468

[ref23] Hansen P. G. Jespersen A. M. (2013). Nudge and the manipulation of choice. A framework for the responsible use of the nudge approach to behaviour change in public policy. Eur. J. Risk Regul. 4, 3–28. doi: 10.1017/S1867299X00002762

[ref24] Harbers M. C. Beulens J. W. Rutters F. de Boer F. Gillebaart M. Sluijs I.. (2020). The effects of nudges on purchases, food choice, and energy intake or content of purchases in real-life food purchasing environments: a systematic review and evidence synthesis. Nutr. J. 19, 1–27. doi: 10.1186/s12937-020-00623-y 32943071 PMC7500553

[ref25] Hess J. M. Jonnalagadda S. S. Slavin J. L. (2016). What is a snack, why do we snack, and how can we choose better snacks? A review of the definitions of snacking, motivations to snack, contributions to dietary intake, and recommendations for improvement. Adv. Nutr. 7, 466–475. doi: 10.3945/an.115.009571 27184274 PMC4863261

[ref26] Hooge I. T. Camps G. (2013). Scan path entropy and arrow plots: capturing scanning behavior of multiple observers. Front. Psychol. 4:996. doi: 10.3389/fpsyg.2013.00996 24399993 PMC3872074

[ref27] Huitink M. Poelman M. P. van den Eynde E. Seidell J. C. Dijkstra S. C. (2020). Social norm nudges in shopping trolleys to promote vegetable purchases: a quasi-experimental study in a supermarket in a deprived urban area in the Netherlands. Appetite 151:104655. doi: 10.1016/j.appet.2020.104655 32247896

[ref28] Kahneman D. (2003). Maps of bounded rationality: psychology for behavioral economics. Am. Econ. Rev. 93, 1449–1475. doi: 10.1257/000282803322655392

[ref29] Keren G. Schul Y. (2009). Two is not always better than one: a critical evaluation of two-system theories. Perspect. Psychol. Sci. 4, 533–550. doi: 10.1111/j.1745-6924.2009.01164.x 26161732

[ref30] Kerr M. A. McCrorie T. A. Rennie K. L. Wallace J. M. Livingstone M. B. E. (2010). Snacking patterns according to location among Northern Ireland children. Int. J. Pediatr. Obes. 5, 243–249. doi: 10.3109/17477160903271963 19878091

[ref31] Kroese F. M. Marchiori D. R. De Ridder D. T. (2016). Nudging healthy food choices: a field experiment at the train station. J. Public Health 38, e133–e137. doi: 10.1093/pubmed/fdv096 26186924

[ref32] Larson N. Story M. (2013). A review of snacking patterns among children and adolescents: what are the implications of snacking for weight status? Child. Obes. 9, 104–115. doi: 10.1089/chi.2012.0108 23470091

[ref33] Loewenstein G. Bryce C. Hagmann D. Rajpal S. (2015). Warning: you are about to be nudged. Behav. Sci. Policy 1, 35–42. doi: 10.1353/bsp.2015.0000

[ref34] Marchiori D. R. Adriaanse M. A. De Ridder D. T. D. (2017). Unresolved questions in nudging research: putting the psychology back in nudging. Soc. Personal. Psychol. Compass 11:e12297. doi: 10.1111/spc3.12297

[ref35] Marteau T. M. Ogilvie D. Roland M. Suhrcke M. Kelly M. P. (2011). Judging nudging: can nudging improve population health? Br. Med. J. 342:d228. doi: 10.1136/bmj.d228 21266441

[ref36] Melnikoff D. E. Bargh J. A. (2018). The mythical number two. Trends Cogn. Sci. 22, 280–293. doi: 10.1016/j.tics.2018.02.001 29571664

[ref37] Michaelsen P. Johansson L. Hedesström M. (2021). Experiencing default nudges: autonomy, manipulation, and choice-satisfaction as judged by people themselves. Behav. Public Policy, 1–22. doi: 10.1017/bpp.2021.5

[ref38] Myhre J. B. Løken E. B. Wandel M. Andersen L. F. (2015). The contribution of snacks to dietary intake and their association with eating location among Norwegian adults–results from a cross-sectional dietary survey. BMC Public Health 15, 1–9. doi: 10.1186/s12889-015-1712-7 25888253 PMC4409996

[ref39] Otterbring T. Wastlund E. Gustafsson A. Shams P. (2014). Vision (im) possible? The effects of in-store signage on customers’ visual attention. J. Retail. Consum. Serv. 21, 676–684. doi: 10.1016/j.jretconser.2014.05.002

[ref40] Papies E. K. Potjes I. Keesman M. Schwinghammer S. Van Koningsbruggen G. M. (2014). Using health primes to reduce unhealthy snack purchases among overweight consumers in a grocery store. Int. J. Obes. 38, 597–602. doi: 10.1038/ijo.2013.136 PMC3982213 23887063

[ref41] Schlinkert C. Gillebaart M. Benjamins J. Poelman M. P. De Ridder D. (2020). Snacks and the city: unexpected low sales of an easy-access, tasty, and healthy snack at an urban snacking hotspot. Int. J. Environ. Res. Public Health 17:7538. doi: 10.3390/ijerph17207538 33081280 PMC7589805

[ref42] Schmidt A. T. Engelen B. (2020). The ethics of nudging: an overview. Philos Compass 15:e12658. doi: 10.1111/phc3.12658

[ref43] Steffel M. Williams E. F. Pogacar R. (2016). Ethically deployed defaults: transparency and consumer protection through disclosure and preference articulation. J. Mark. Res. 53, 865–880. doi: 10.1509/jmr.14.0421

[ref44] Sunstein C. R. (2017). Nudges that fail. Behavioural. Public Policy 1, 4–25. doi: 10.1017/bpp.2016.3

[ref45] Szaszi B. Palinkas A. Palfi B. Szollosi A. Aczel B. (2018). A systematic scoping review of the choice architecture movement: toward understanding when and why nudges work. J. Behav. Decis. Mak. 31, 355–366. doi: 10.1002/bdm.2035

[ref46] Thaler R. H. Sunstein C. R. (2008). Nudge: improving decisions about health, wealth, and happiness. New Haven, CT: Yale University Press.

[ref47] Van der Laan L. N. Papies E. K. Hooge I. T. Smeets P. A. (2017). Goal-directed visual attention drives health goal priming: an eye-tracking experiment. Health Psychol. 36:82. doi: 10.1037/hea0000410 27631308

[ref48] Van Gestel L. C. Adriaanse M. A. De Ridder D. T. D. (2020). Do nudges make use of automatic processing? Unraveling the effects of a default nudge under type 1 and type 2 processing. Comprehensive Results in Social Psychology. 5, 4–24. doi: 10.1080/23743603.2020.1808456

[ref49] Van Gestel L. C. Kroese F. M. De Ridder D. T. D. (2018). Nudging at the checkout counter: a longitudinal study of the effect of a food repositioning nudge on healthy food choice. Psychol. Health 33, 800–809. doi: 10.1080/08870446.2017.1416116 29252010

[ref50] Venema T. A. Kroese F. M. Benjamins J. S. De Ridder D. T. (2020). When in doubt, follow the crowd? Responsiveness to social proof nudges in the absence of clear preferences. Front. Psychol. 11:1385. doi: 10.3389/fpsyg.2020.01385 32655456 PMC7325907

[ref51] Venema T. A. Kroese F. M. De Vet E. De Ridder D. T. (2019). The one that I want: strong personal preferences render the center-stage nudge redundant. Food Qual. Prefer. 78:103744. doi: 10.1016/j.foodqual.2019.103744

[ref52] Wachner J. Adriaanse M. A. De Ridder D. T. D. (2021). The effect of nudges on autonomy in hypothetical and real life settings. PLoS One 16:e0256124. doi: 10.1371/journal.pone.0256124 34428254 PMC8384220

[ref53] Wästlund E. Otterbring T. Gustafsson A. Shams P. (2015). Heuristics and resource depletion: eye-tracking customers’ in situ gaze behavior in the field. J. Bus. Res. 68, 95–101. doi: 10.1016/j.jbusres.2014.05.001

[ref54] World Health Organization (2021a). Controlling the global obesity epidemic. Available at: https://www.who.int/activities/controlling-the-global-obesity-epidemic (Accessed August 9, 2021).

[ref55] World Health Organization (2021b). Obesity and overweight. Available at: https://www.who.int/news-room/fact-sheets/detail/obesity-and-overweight (Accessed August 9, 2021).

