# Supplementary material for: The role of attention and health goals in nudging healthy food choice
Source: Front Psychol. 2023 Oct 19;14:1270207. doi: 10.3389/fpsyg.2023.1270207 (PMC10620715; doi:10.3389/fpsyg.2023.1270207)
Supplement: Supplementary file 1 [file Data_Sheet_1.PDF]

Supplementary material

The role of attention and health goals in nudging healthy food choice

Marleen Gillebaart[1*], Stephanie S.A.H. Blom[1], Jeroen S. Benjamins[1,2], Femke de Boer[1], and Denise T.D. De Ridder[1]

[1]Department of Social, Health and Organizational Psychology, Utrecht University, Utrecht, The Netherlands
[2]Department of Experimental Psychology, Utrecht University, Utrecht, The Netherlands

*** Correspondence:**
Marleen Gillebaart
M.Gillebaart@uu.nl

Method

Control measures

Hunger. To measure how hungry participants were, we asked "*How hungry are you right now?",* answered on a scale from 0 (*not hungry at all*) to 10 (*very hungry*) (as in Van Gestel et al., 2018).

BMI. Participants reported their weight in kilograms and their length in meters. Body Mass Index (BMI) was calculated (Dutch Nutrition Centre, 2019), and categorized into: no overweight (BMI < 25) and overweight (BMI ≥ 25).

General snack habit. Questions derived from the Self Report Habit Index (SHRI) questionnaire (Verplanken & Orbell, 2003) were used to measure snack habit. The questions: *"Buying a snack is something I often do"*, *"Buying a snack is something that would cost me effort not to do*", "*Buying a snack belongs to my routine"*, and *"Buying a snack is typical something for me"* were answered on a scale from 1 (*totally disagree*) to 7 (*totally agree*). The scale showed to have a good internal consistency in the current study (α = .879).

Store familiarity. Two questions were asked to indicate participants' store familiarity. First, we asked "*How often do you do your groceries in this supermarket?*", with answer categories 1 (often, [...] times per week), 2 (sometimes, [...] times per month), and 3 (almost never, namely: […]). Second, we asked: "*For how long have you been visiting this supermarket?*", with answer categories 1 (less than two months), 2 (two to six months), and 3 (more than six months).

Results

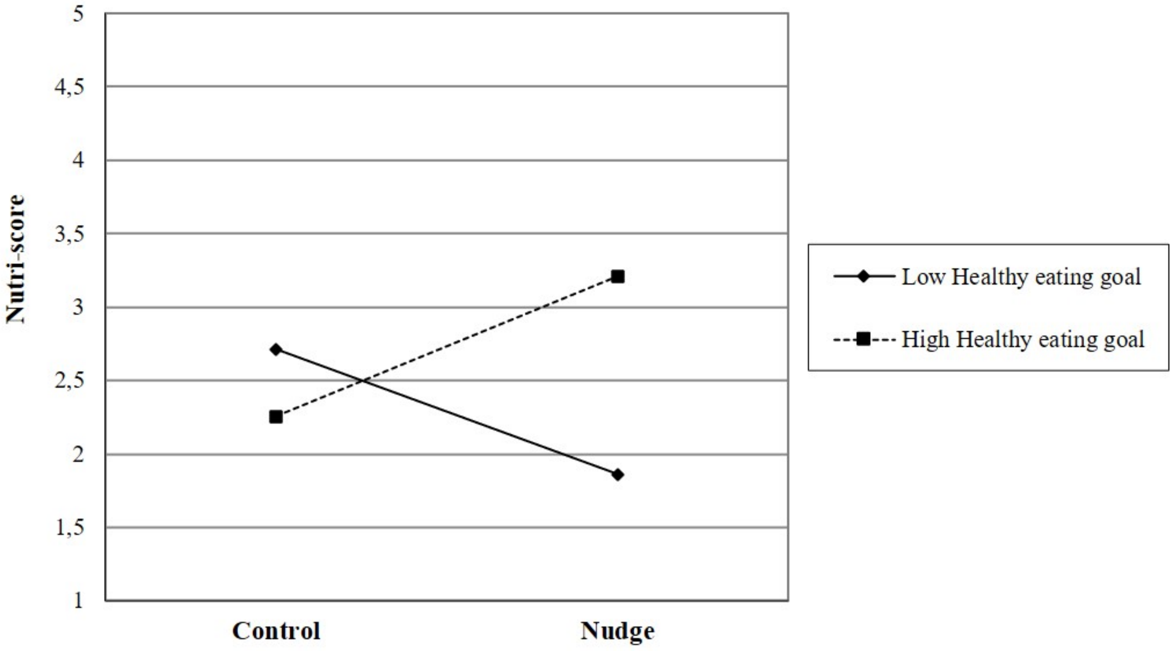

Figure 2.

Visual depiction of the simple slope analysis probing the interaction between experimental condition and healthy eating goals (low healthy eating goal being 1SD below the mean, high healthy eating goal being 1SD above the mean) from the regression analyses on explicit attention,

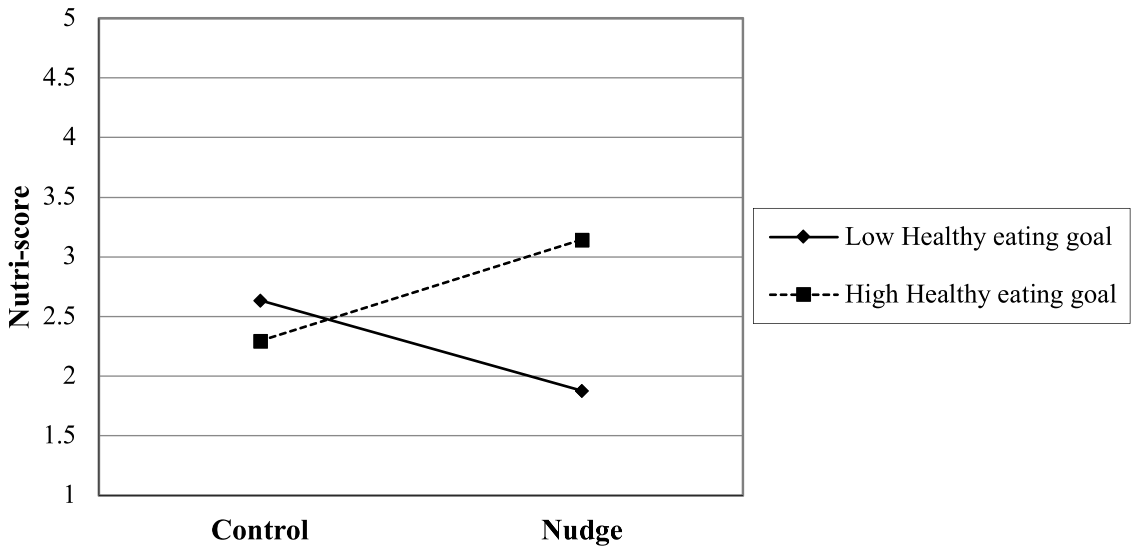

Figure 3.

Visual depiction of the simple slope analysis probing the interaction between experimental condition and healthy eating goal (low healthy eating goal being 1SD below the mean, high healthy eating goal being 1SD above the mean) from the regression analyses on implicit attention,

References

Aiken, L. S., West, S. G., & Reno, R. R. (1991). *Multiple regression: Testing and interpreting interactions*. Sage.

Dutch Nutrition Centre (Voedingscentrum) (n.d.). BMI berekenen. Retrieved from: https://www.voedingscentrum.nl/bmi

European Commission (n.d.). The European Qualifications Framework. https://europa.eu/europass/en/european-qualifications-framework-eqf. Accessed 12 August 2021.

Verplanken, B., & Orbell, S. (2003). Reflections on past behavior: a self-report index of habit strength 1. *Journal of Applied Social Psychology*, *33*(6), 1313-1330.
